# A231 COLORECTAL CANCER PROVINCIAL SCREENING OPTIMIZATION USING GUT MICROBIOME AS BIOMARKER

**DOI:** 10.1093/jcag/gwab049.230

**Published:** 2022-02-21

**Authors:** I Laforest-Lapointe

**Affiliations:** Biologie, Universite de Sherbrooke Faculte des Sciences, Sherbrooke, QC, Canada

## Abstract

**Background:**

Colorectal cancer (CRC) is a disease easy to cure but quite difficult to detect. Because of that, the mortality rate of CRC is among the highest in the world. The current way to clinically detect CRC is by mean of immunochromatographic fecal occult blood test (iFOBT). If the test is positive, the patient undergoes a colonoscopy to confirm the presence and stage of CRC. However, iFOBT tests are affected by a high rate of false positives. In addition, colonoscopy preparation and intervention have major drawbacks on patients’ health. It is thus important to reduce the false negative rate of iFOBT.

**Aims:**

In this study, we aim to find microbiome biomarkers that could reduce the rate of false positives, quantify microbiome composition as CRC worsens, and finally improve iFOBT accuracy by adding microbial biomarkers and CRC-related human genes to the detection for CRC.

**Methods:**

To do that, 1048 fecal samples were collected with iFOBT kit (OC-Auto® FIT test Kit) then send to the CHUS (Centre Hospitalier de l’Université de Sherbrooke). Sex, age and health status (healthy with “no blood”; false positive: FP; adenoma, adenocarcinoma) were collected for 952 samples. Those samples come from healthy patient with “no blood” (no or below 175 ng/mL of blood in sample), FP patients (healthy but with a concentration above 175 ng/mL of blood), 387 patients with adenoma and 52 patients with adenocarcinoma. The community microbial DNA were extracted from samples by using QIAGEN QIAmp Fast DNA stool mini-kit. An amplification of the V4 region of the 16S rRNA gene were done by using the primers 515 F and 806R, then sequenced on a MiSeq platform.

**Results:**

First, taxonomy was assigned to each ASV (Amplicon Sequence Variant) with Silva. Then ASVs were parsed through ten different machine learning algorithms to assess if the microbiome can increase the power of prediction (sensitivity and specificity) of CRC by comparing FP vs adenoma and FP vs adenocarcinoma by generating AUC-ROC (Area Under the Curve-Receiver Operating Characteristics) curves using only the sex, age and occult blood concentration, and then adding the ASVs community.

Preliminary results show that the power of prediction of CRC with only sex, age and occult blood concentration have an AUC-ROC curves varying between 0.53–61 when comparing FP vs adenoma, and an AUC-ROC curves varying between 0.56–0.74 when comparing FP vs adenocarcinoma. And when adding the community matrix, we see that the AUC-ROC curves comparing FP vs adenoma are varying between 0.48–0.68; and when comparing FP vs adenocarcinoma, the AUC-ROC curves are varying between 0.51–0.98.

**Conclusions:**

In the future, we hope to explore the possibility of using this machine learning protocol to determine the stage of CRC disease and reduce the need for FP colonoscopies.

**Funding Agencies:**

CIHRCanada Research Chair

